# Digital Media Coverage of Respiratory Syncytial Virus-Related News in India: Mixed Methods Content Analysis of Disease Burden and Intervention

**DOI:** 10.2196/70322

**Published:** 2025-08-05

**Authors:** Rhythm Hora, Arindam Ray, Amrita Kumari, Rashmi Mehra, Amanjot Kaur, Syed F Quadri, Bodhisatwa Ray, Seema Singh Koshal, Shyam Kumar Singh, Abida Sultana, Arup Deb Roy

**Affiliations:** ^1^John Snow India, Plot No.5 & 6, LSC Shopping Complex, Nelson Mandela Marg, Vasant Kunj, New Delhi, 110070, India, 91 9368096447; 2 Gates Foundation, Seattle, WA, United States; 3 University of Exeter, Exeter, United Kingdom

**Keywords:** vaccine content analysis, respiratory syncytial virus, RSV, respiratory syncytial virus vaccine, digital media, public health

## Abstract

**Background:**

Respiratory syncytial virus (RSV) is a leading cause of lower respiratory tract infections in children younger than 5 years of age. Given the high morbidity and mortality associated with RSV in India, the introduction of a vaccine against RSV will potentially reduce the disease’s burden. However, vaccine acceptance is influenced by public perception, which is shaped by information disseminated through media sources. This study aims to explore the landscape of RSV–related news coverage in India’s digital media.

**Objective:**

This study aims to conduct a comprehensive content analysis to explore the landscape of RSV–related news coverage in India’s digital media.

**Methods:**

Media content analysis was retrospectively conducted by a digital search for all related news pieces in the trustworthy brands of 4 trusted newspapers (Hindustan Times, The Hindu, The Indian Express, and The Times of India) and 3 news channel websites (India Today, NDTV news, and News 18), between November 1, 2022, and October 31, 2023. A total of 58 news pieces were retrieved using selected keywords, with inclusion criteria encompassing English-language news pieces with RSV–specific content. Two reviewers compiled, coded, and analyzed the content. Quantitative data were analyzed descriptively, while qualitative content analysis assessed the emotional tone and sentiment of the pieces.

**Results:**

The findings revealed significant digital media coverage on RSV infection and the potential vaccines. The majority of news pieces (53/58, 91%) discussed RSV signs and symptoms, with 64% (37/58) addressing the disease severity and 36% (21/58) highlighting its seasonal surge. However, only 5% (3/58) focused on diagnostic aids. Additionally, 41% (24/58) of news pieces discussed RSV in the context of COVID-19. Regarding the vaccine, 29% (17/58) of news pieces mentioned it, with 26% (15/58) highlighting manufacturers such as Pfizer and GlaxoSmithKline (GSK). Positive sentiment was found in 35% (20/58) of news pieces, while 43% (25/58) exhibited negative sentiment, often related to the disease burden and severity. Emotional tone analysis revealed that 74% (43/58) of news pieces contained emotional elements, with 58% (25/43) expressing negative emotions (eg, concern and anxiety), particularly about hospitalizations and deaths. In contrast, a positive tone was emulated in the frequent mentions of the RSV vaccines as safe, effective, and approved.

**Conclusions:**

The analysis revealed significant coverage of RSV–related news in India’s digital media, with a focus on disease severity and hospitalizations. While positive sentiment was expressed in coverage of the RSV vaccine, negative sentiments dominated discussions on the disease burden. However, considering the limited number of news pieces, the study highlights the need for improved media coverage to raise awareness about the disease and its preventive strategies. Further research should explore the implications of the overlap between RSV and COVID-19 in media coverage and the limited focus on RSV diagnostics, with a focus on understanding how these factors impact public health outcomes.

## Introduction

Respiratory syncytial virus (RSV) stands as a leading cause of lower respiratory tract infections in children younger than 5 years of age, posing a significant public health challenge worldwide [1,2]. The estimates for 2019 show 33 million episodes of RSV-associated lower respiratory infection in this vulnerable age group each year worldwide, with a striking 95% of these cases occurring in lower-middle-income countries [3]. This underscores the disproportionate burden faced by these regions in managing RSV-related health challenges.

In India alone, the situation is terrible, with the National Health Portal report (2019) stating 41.9 million cases of respiratory infections in 2018 [4]. These infections not only led to a significant number of hospitalizations but also contributed to 0.003 million fatalities during that year [4].

To control the growing burden of RSV, adopting preventive measures such as vaccination is sought to be the most promising and cost-effective public health intervention [5]. RSV vaccines can significantly reduce the incidence and severity of infectious diseases, preventing hospitalizations, reducing health care costs, and saving lives [6]. However, the success of these vaccination programs goes beyond the mere availability of vaccines and hinges significantly on community acceptance, which is greatly influenced by the information disseminated [7]. Moreover, the community perception is largely influenced by the various sources of information the individuals come across, such as health care providers, print media, digital media, and social media [5,8,9]. Recent research indicates the significant role of media sources in shaping or influencing people’s perceptions and attitudes toward public health services [5,10,11]. Concurrently, digital media, including digital news platforms, have emerged as prime avenues for the rapid and widespread dissemination of information related to diseases or infections and their preventive measures [11-13]. Given this critical role, it is imperative or critical to assess how RSV-related news is presented in the digital landscape, particularly in a country like India where media consumption patterns are rapidly evolving [14-17]. This context underscores the critical need for a comprehensive analysis of news coverage related to RSV in India’s digital media. Therefore, this study aims to conduct an in-depth content analysis to assess the portrayal of RSV-related information in news coverage across digital media platforms in India.

## Methods

The study was done in accordance with the SRQR (Standards for Reporting Qualitative Research) guidelines (Checklist 1) [18,19].

### Study Design

Media content analysis was retrospectively conducted by digital search for news pieces published on the leading newspapers’ websites and news channel websites appearing over 1 year (November 1, 2022, to October 31, 2023). National newspapers and news channel websites were included in this study due to their wide audience reach, credibility, consistency, and ability to effectively capture the dominant narratives and trends that influence public opinion. Furthermore, the 1-year time frame was selected to capture relevant trends and events, ensuring a comprehensive analysis of current developments. Additionally, this period offered a balanced perspective on seasonal variations and emerging patterns in RSV-related news coverage within the digital media landscape.

### Data Extraction Strategy

This study used conceptual content analysis of the most trustworthy brands of national newspapers and news channel websites [20,21]. Based on the brand trust scores and SCImago media rankings, 4 trusted national newspapers (Hindustan Times, The Hindu, The Indian Express, and The Times of India) and 3 trusted national news channel websites (India Today, NDTV News, and News 18) in cyberspace were selected [20,21]. A standardized extraction method using the selected keywords was prepared for retrieving the desired information. The dataset included all the news pieces retrieved after entering the selected keywords: “RSV” or “RSV vaccine,” and “RSV” and “vaccine.” Only news pieces containing these exact words were extracted. The search was limited only to news pieces in English with all RSV-specific content, whether in the Indian or global context. Focusing on mainstream media in a dominant language (eg, English) was chosen to ensure consistency in content, audience, and reach to obtain more reliable conclusions. This resulted in the retrieval of 62 news pieces from the newspapers and news channel websites included in the study. News pieces were excluded if they were duplicates (news pieces published around the same time, with the same headline and text in the same publication). The news pieces in the vernacular language were excluded from the study due to the potential for inaccuracies or misinterpretations arising from the researchers’ limited understanding of regional languages. Besides, no editorials or feature stories were included in the study, as they are subjective opinion pieces that could skew the findings, which are focused on objective reporting. Furthermore, health blogs and independent media were not included in the study to maintain a focus on reliable, widely recognized sources that influence mainstream discourse. After excluding the irrelevant news pieces, 58 news pieces were yielded for analysis.

### Data Analysis

To begin with, the news pieces were numbered for ease of reference. Later, the data were analyzed in two steps: first, a quantitative analysis of the news pieces’ characteristics and the nature of the information presented, including the sentimental and emotional tonality. Later, this was followed by the qualitative analysis to further account for the sentimental and emotional content in the news pieces.

For the quantitative analysis, a codebook was developed through an iterative process by reviewing a few arbitrarily selected news pieces to identify predominant themes and generate codes. In total, 10% (n=6) of the news pieces were coded independently by two skilled coders (RH and RM) using NVivo (version 14; Lumivero) qualitative data analysis software. Intercoder reliability was measured to ensure agreement between the coders while coding the same dataset. The reliability criterion set for this study was κ _0.60, which is considered ample according to recommended sources [22]. Disagreements were discussed with another author (AKaur), and consensus was reached. The codebook generated captured information on 4 main categories: information on RSV (including diagnostics, seasonal surge, and co-mention with COVID-19), information on RSV vaccine (including the manufacturers, potential recipients, approval status, and concerns around efficacy or safety), sentiment in title or content, and emotional tone. The data were statistically analyzed using SPSS (version 21.0; IBM Corp) and results were expressed in numbers and percentages.

For qualitative analysis, in-depth content analysis of the news pieces was done to thematically explore sentiment analysis (negative, neutral, or positive) and emotion analysis (record the emotional tone). In this context, negative sentiment refers to expressions or views that convey unfavorable attitudes, disapproval, sadness, or dissatisfaction toward a subject. On the other hand, positive sentiment refers to expressions or views that convey favorable attitudes, approval, happiness, or satisfaction toward the subject. Additionally, emotional tone conveys the overall emotive quality expressed in a piece of text, reflecting the author’s or speaker’s feelings toward the subject matter. Emotional tone was assessed using methods like the NRC Emotion Lexicon, which associates English words with 8 core emotions (anger, fear, anticipation, trust, surprise, sadness, joy, and disgust). For instance, words such as “concern” or “anxiety” were associated with emotions like fear or sadness, while terms like “hope” or “joy” were connected to positive emotions [23].

This analysis used the initially generated codebook to code all news pieces using NVivo. The coding was primarily done to delineate content specific to the sentiment conveyed in the title or content of all news pieces. Coded data were then read and reread to arrive at a consensus.

### Ethical Considerations

No ethical approval was sought for the study, as it used publicly available data of a nonidentifiable nature.

## Results

A total of 58 news pieces were included in the analysis. Our findings underscore the various media sources (the media source and the news source), the nature of the information presented, and the emotional tones conveyed in the reporting.

### Characteristics of News Stories

#### Media Source

**Figure 1.**
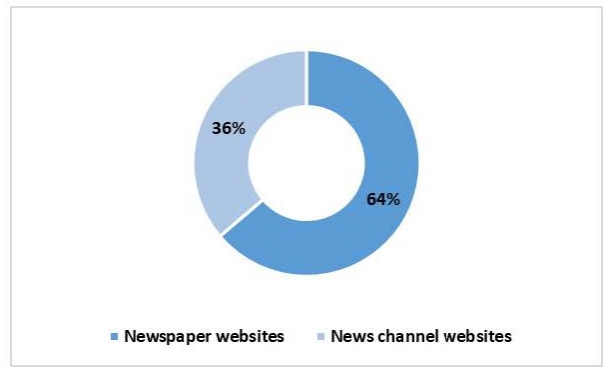
Comprehensive overview of the sources of retrieved news pieces in the digital media landscape (N=58).

Of the 58 news pieces included in the analysis, 37 (64%) were identified from the leading national newspapers and 21 (36%) from the leading news channel websites selected for the study (Figure 1).

#### News Source

The news pieces (N=58) relied on various sources (Table 1); however, in almost one-third, that is, 20/58 (35%), the news source was the news or web desk (Hindu Bureau, Times News Network (TNN), and Express-News). The other most common news source was Reuters (n=9, 15%).

**Table 1.** Description of the identified news sources featured in the retrieved digital media news (N=58).

News source	Frequency (N=58), n (%)
Press Trust of India (PTI)	4 (7)
Reuters	9 (15)
Agence France-Presse (AFP)	5 (9)
Asian News International (ANI)	2 (3)
Associated Press (AP)	4 (7)
News desk or web desk (Hindu bureau, TNN^a^, and Express News)	20 (35)
Others: Individual person name or lifestyle desk or local circles	14 (24)

a TNN: Times News Network

### Nature of Information in News Pieces

#### Information on RSV

Of the total news pieces (N=58) retrieved, 53 (91%) news pieces had a detailed description of the signs and symptoms of RSV infection, with 37 (64%) mentioning the severity of the infection and 21 (36%) discussing the seasonal surge of the RSV infection (Table 2). Besides, 24 (41%) news pieces reporting on the COVID-19 infection also discussed the RSV infection. In total, 1 (2%) news piece stated the modes of transmission of RSV infection, while 12 (21%) mentioned the potential complications and the modes of prevention of RSV infection. Only 3 (5%) news pieces discussed the diagnostic aids available for detecting RSV infection.

**Table 2.** News stories on RSV^a^ infection and RSV vaccine featured in the digital media landscape.

Categories and codes identified		Frequency (N=58), n (%)
RSV		
Signs and symptoms of disease		53 (91)
RSV burden (epidemiology)		10 (17)
Modes of transmission		14 (24)
Severity of disease		37 (64)
Potential complications		12 (21)
Modes of prevention		12 (21)
RSV diagnostics		3 (5)
Seasonal RSV surge		21 (36)
RSV and COVID-19		24 (41)
RSV vaccine		
Overview about vaccine		17 (29)
Vaccine products and manufacturers		15 (26)
Vaccine approval status		8 (14)
Vaccine trials		5 (9)
Potential recipients of vaccine		15 (25)
Vaccine efficacy		9 (15)
Vaccine safety		6 (10)
Cost or price		2 (3)
Sentiment in title or content		
Positive		20 (35)
Neutral		13 (22)
Negative		25 (43)
Emotional tone		
Yes		43 (74)
No		15 (26)

a RSV: respiratory syncytial virus.

#### Information on RSV Vaccine

Out of all the news pieces (N=58) included in the study, 17 (29%) pieces had a mention of the RSV vaccine. In total, 15/ 58 (26%) news pieces had a clear mention of the globally available vaccine products and their manufacturers (Table 1) with 7 (47%) referencing only the Pfizer vaccine product (Abrysvo) and 5 (33%) of the GSK vaccine product (Arexvy), and 1 mentioned AstraZeneca and Sanofi’s product (Nirsevimab). Only 2 news pieces reported on both the leading vaccine manufacturers (Pfizer and GSK) and their products, and that they competed with each other.

Furthermore, 15 (26%) news pieces specifically mentioned the potential recipients of the RSV vaccine. Of the 15 news pieces, 9 (60%) referenced older adults aged 60 years and older as potential recipients, while 6 (40%) news pieces mentioned pregnant women as potential recipients (Table 2).

Besides, 9 (15%) and 6 (10%) news pieces discussed the RSV vaccine efficacy and safety, respectively. Only 2 (3%) new pieces covered the cost/ price of the RSV vaccine.

### Sentiment in Title or Content

Most (n=25, 43%) of the news pieces had a negative sentiment in the title or content. However, 20 (35%) news pieces had a positive and 13 (22%) had a neutral sentiment in the title or content (Table 2). Furthermore, in all 17 news pieces (Table 2) that reported the RSV vaccine, there was a positive sentiment in the title and the content.

### Emotional Tone

The emotional tone was also analyzed for all the news pieces (n=58). Nearly 43 of 58 (74%) of the news pieces contained emotional elements either around the RSV infection or the RSV vaccine. However, only 25 of 43 (58%) news pieces stated negative emotions that were entirely related to RSV disease (Table 2).

### Qualitative Content Analysis

#### Sentiment in Title or Content

##### Negative Sentiments Toward the Vaccine

Among 25 of 58 (43%) news pieces with negative sentiments, the most recurring negative sentiment was the increased burden of respiratory illnesses and RSV infection, with its seasonal surge found in 21 of 25 news pieces (Table 2). For example, the medical director of Bharati Vidyapeeth Medical Hospital and Research Center said

*Viruses like influenza and parainfluenza are now the dominant ones. Both in children and adults, we are witnessing an increasing incidence of respiratory viral illnesses, as the humidity levels have been quite high. The admission pattern across hospitals shows that ICU admissions have increased by nearly 25%‐30%*.

[News piece 27]

The other negative theme that emerged was the severity of the infection, resulting in hospitalizations and deaths. For example

*“This was the hardest week of my life,” wrote American standup comedian and actor Amy Schumer on Instagram when her three-year-old son was admitted to the hospital, adding that her son was rushed to ER and admitted for RSV.*

[News piece 42]

The negative theme related to the RSV vaccine was a limitation in vaccine introduction and implementation, owing to the cost of the vaccine (2 news pieces). News piece 52 mentions that *“*the vaccine would be priced above $120 per shot, provided, expected soon, shows that it offers protection for two RSV seasons.”

##### Positive Sentiments Toward the Vaccine

The most recurring positive theme was the approval of vaccines (17 of the 20 news pieces with positive sentiment) to prevent RSV-related disease burden. Almost all these news pieces assured that the approved vaccines are safe and effective, with common and mild side effects. Referring to the declaration by the medical director of the National Foundation for Infectious Diseases, news piece 29 mentioned, *“*This is a great first step ... to protect older persons from serious RSV disease*.*”

Another positive theme (3 of 20 news pieces) was continued screening and diagnostics for RSV and other viral or flu packages. For example, *“*Tamil Nadu to continue fever screening!*”* is mentioned in the title of news piece 31.

### Emotional Tone

Of the total news pieces (n=43) with emotions included, negative emotions were reflected in 25 (58%) news pieces. The negative emotions expressed included adjectives such as concern, anxiety, agony, and grief due to disease burden, hospitalizations, severity of infection, and deaths. These negative emotive patterns were chiefly around the RSV infection only. For example, citing the statement of a pediatrician, one news piece mentioned:

*We are seeing a significant number of pediatric cases in the outpatient department, testing positive for RSV. While RSV has the same symptoms as influenza, including cold, cough, and fever in infants, wheezing is also seen in newborns. Some are coming with bronchiolitis and have to be put on respiratory support and ICU care.*

[News piece 24]

## Discussion

### Principal Findings and Interpretation

The burden of RSV infection in the younger than 5 years age group worldwide necessitates the need to unveil increasing concerns around a booming public health challenge. As digital media plays a crucial role in communicating public health issues and policy information, this study explored the news content related to RSV in the trusted brands of newspapers and news channels in the digital environment.

The study findings revealed that digital news media have provided quite a reasonable amount of information on the signs and symptoms, modes of transmission and prevention, and severity of RSV infection. This is crucial for generating public awareness and could potentially drive early recognition and treatment [24]. However, less information on diagnostic aids (n=3, 5%) in the news pieces reflects a notable gap in comprehensive health communication and management. Previous literature also highlights that a thorough understanding of information on diagnostics makes a ground for bureaucratic interactions and influences public policy formulations [25,26]. Further, the overlap of RSV and COVID-19 across news pieces (n=24, 41%) suggests that the COVID-19 pandemic continues to influence the narratives surrounding respiratory illnesses. Discussions in the existing literature emphasize that other respiratory diseases should not be overshadowed, in light of the COVID-19 pandemic, as the consequences could be dramatic [27].

This study also found a decent amount of information on vaccines available to prevent RSV infection, as a promising strategy to alleviate RSV-related morbidity and mortality. Additionally, the news pieces have discussed the vaccine manufacturers and potential recipients of the vaccine, including the approval status of the vaccines. Besides, there have been deliberations in the news pieces regarding the efficacy or safety of the vaccine. A content analysis of newspaper coverage of the COVID-19 pandemic in 2021 demonstrated that updates on the progress of vaccines, in general, might often provide better hope to the people and bring them psychological harmony [28].

Furthermore, our analysis showed that most news coverage expressed negative opinions regarding the burden of respiratory illnesses, including RSV infections and associated hospitalizations. This aligns with previous research indicating that media reporting often emphasizes negative aspects of public health crises, which can heighten public anxiety [29,30]. Only 2 news pieces discussed the cost of the RSV vaccine, which was a limitation in vaccine introduction and implementation. Moreover, negative emotions elicited by news pieces, such as personal narratives from affected families, amplify the emotional tone. This is consistent with other studies demonstrating that people often respond more strongly to the negative elements in their environment, reflecting the media’s power to shape public perception [28,31]. Besides, the approval of safe and efficacious RSV vaccines, which may be viewed as a beacon of optimism, contributed to the positive tone of all news pieces reporting on the RSV vaccine. Previous studies mentioned that media narratives can drive policy-level solutions and influence public willingness to accept the vaccine [28,32].

### Limitations and Recommendations

The study also has some limitations. Although the news pieces over the past 1 year had RSV coverage, considering the time frame included in the study, the number of news pieces retrieved was limited. Furthermore, this analysis focused on news pieces but did not include editorials or feature stories that may have contained additional information. Besides, news pieces in the vernacular language were not explored for this study. Another potential limitation of the study was that the data source was not exhaustive. Thus, there may be relative under or overestimation of data as the study only selected newspapers and news channel websites for data collection for practical reasons, while other forms of media, including digital media (including social media), electronic media (including television, radio, and cinema), and magazines, were not used.

This study did not examine the implications of the overlap between RSV and COVID-19 in media coverage, nor the limited focus on RSV diagnostics. Future research to explore how this overlap may cause confusion, affect risk perceptions, and influence health priorities, as well as how the gap in coverage of RSV diagnostics impacts public awareness and health outcomes, is sought. Furthermore, the study recommends that policy makers enhance public health communication and encourage media outlets to rely on credible health sources for clearer, more consistent reporting, especially during overlapping health crises, to improve public understanding and reduce confusion.

### Conclusions

This study sought to analyze the portrayal of RSV-related news within the digital media landscape in India. The study highlighted a substantial presence of RSV and RSV-related information in digital media. Content analysis of screened news pieces revealed that negative sentiments were often found in news pieces capturing the burden of respiratory illnesses, including RSV infections and associated hospitalizations. On the other hand, there was a consistent positive emotion in the news pieces around the approval of safe and effective vaccines to prevent RSV-related disease burden. As the media plays a crucial role in communicating public health and policy information, the study elicits the need for improved media coverage. This will aid in communicating appropriate messages and generating awareness about the disease, including its preventive strategies. In addition, the study highlights the need for improved public health communication by policy makers, particularly during overlapping health crises. Furthermore, a larger study can be undertaken to include news in the vernacular languages, especially in the Indian context. Future research to explore the implications of the overlap between RSV and COVID-19, along with the gap in coverage of RSV diagnostics on public health outcomes, is recommended.

## Supplementary material

10.2196/70322 Checklist 1 SRQR (Standards for Reporting Qualitative Research) checklist.

## Abbreviations

RSV: respiratory syncytial virus
SRQR: Standards for Reporting Qualitative Research
